# Sequencing and phylogenetic analysis of the *stn* gene of *Salmonella* species isolated from different environmental sources at Lake Qarun protectorate: The role of migratory birds and public health importance

**DOI:** 10.14202/vetworld.2021.2764-2772

**Published:** 2021-10-26

**Authors:** Hanan S. Khalefa, Zeinab S. Ahmed, Fatma Abdel-Kader, Eman M. Ismail, Esraa A. Elshafiee

**Affiliations:** 1 Department of Veterinary Hygiene and Management, Faculty of Veterinary Medicine, Cairo University, Giza 12211, Egypt; 2 Department of Zoonoses, Faculty of Veterinary Medicine, Cairo University, Giza 12211, Egypt.

**Keywords:** migratory aquatic birds, fish, poultry, *Salmonella*, *stn* gene, Lake Qarun

## Abstract

**Background and Aim:**

*Salmonella* causes most foodborne bacterial illnesses worldwide. It is found in various hosts, including pets, farm animals, and wild animals, as well as the environment. This study aimed to examine the epidemiological relationship between *Salmonella* isolates from aquatic environments and those from other avian hosts.

**Materials and Methods:**

The study examined 12 water samples, 210 aquatic animals, and 45 migratory aquatic bird samples collected from the protected area of Lake Qarun in El-Fayoum Governorate, Egypt, during migration seasons from different waterfowl migration areas (from October 2018 to January 2019). In addition, 45 fecal samples from domestic chickens were collected from the same geographic location from poultry farms. Bacteriological examination and polymerase chain reaction assay of two virulence genes (i.e., *invA* and *stn*) were performed to isolate and identify *Salmonella*.

**Results:**

*Salmonella* was isolated from 58.3% (7/12) of Lake Qarun water samples, 13.3% (6/45) of migratory waterfowl, 6.6% of (3/45) of chickens (*Gallus gallus domesticus*), and 4.3% (3/70) of fish and pooled brine shrimp. In migratory aquatic bird species that were sampled, *Salmonella* were isolated from 23.1% (3/13) of Eurasian coot (*Fulica atra*), 12.5%, (1/8) of green-winged teal (*Anas cardolinesis*), 10% (2/20) of northern shoveler (*Spatula clypeata*), and 0% (0/4) of mallard duck (*Anas platyrhynchos*). In 35 Tilapia, *Salmonella* was isolated by (8.6%) 5.7% of external surfaces, 2.85% from the intestine, and 0% from the muscle. No *Salmonella* was isolated from the 175 brine shrimp samples. Phylogenetic analysis using the *stn* genes of *Salmonella* isolated from the aquatic environment, migratory aquatic birds, and chicken showed a strong association between these isolates. In addition, a higher nucleotide identity percentage was observed between the sequences recovered from migratory aquatic birds and Lake Qarun water samples.

**Conclusion:**

*Salmonella* distribution was confirmed through migratory aquatic birds, based on our phylogeny tree analysis, *Salmonella* considered a likely carrier of zoonotic bacterial pathogens. Furthermore, the close relationship between chicken and fish sequences highlights the scenarios of using chicken manure in fish farms and its public health implications. The presence of *Salmonella* in different environmental sources spotlights the urgent need to control and break down its epidemiological cycle.

## Introduction

*Salmonella* is a highly virulent enteric pathogen but can also be found in various hosts, including pets, farm animals, domesticated animals, and insects [1,2]. Moreover, several recent studies have found that *Salmonella* can be detected in surface waters in several countries worldwide [3,4]. As a result, *Salmonella* has traditionally been viewed as zoonotic bacteria. Recent study has revealed that the environment can be a source of *Salmonella* [5]. The widespread presence of these bacteria in aquatic environments suggests that water serves as a reservoir for *Salmonella*. [6].

Lake Qarun, Egypt’s third-largest lake, has an estimated surface area of approximately 240 km^2^ and is an important wetland for migratory and resident aquatic birds alike [7]. In addition, Lake Qarun is the only enclosed, salty, and highly eutrophic lake in Egypt [8]. The lake is important for Egyptian fisheries, as it results in significant total catches and a wide array of economically valuable species [9]. In spite of Lake Qarun being designated as a protected area (PA) in 1989, it has not been protected from pollution. Among the factors that influence Lake Qarun’s ecosystem are climatic conditions, wastewater discharge, and seepage from cultivated fields nearby. The two main drainages are the El-Batts and El-Wadi, which carry approximately 450 million metric tons of untreated agricultural, industrial, sewage, and domestic effluents to the lake annually from El-Fayoum Province [10-13]. Several environmental problems we face today can be attributed to industrial and agricultural technology. Around this lake, numerous fish farms have been established [14]. Lake Qarun is experiencing increasing water pollution, resulting in significant adverse effects on human health and the aquatic environment. Recently, fish farming has emerged on the southern shores of Lake Qarun. Fishing is performed on the lake by rowing boats and nets, and mining and industrial activities are conducted in the area as well. These contaminants are ultimately receptacles in the environment of the lake. Fish can be exposed to various pollutants through water, food, sediments, and suspended particulates [15].

In winter, Lake Qarun holds large numbers of waterfowl. At least ten species of birds breed here. *Salmonella* serovars are common in both wild and domesticated birds and may exert a significant influence on the epidemiology of salmonellosis in humans and farm animals [16]. The presence of wildlife in the environment, including birds, may influence *Salmonella* populations as well [17].

Chicken manure has been used in semi-intensive fish farming practices. In addition to being directly consumed by fish, they mainly stimulate photosynthetic organisms [18,19]. Because poultry manure is easily soluble and contains a high concentration of phosphorus, it is preferred over other types of animal manure [20]. However, a manure fertilizer poses a potential threat to the aquatic environment and is, thus, considered a hazardous organic matter. Moreover, it represents a great concern for public health because confined farming systems with little water exchange can spread various microorganisms from animal manure [21].

To conduct our study, we targeted *Salmonella* enterotoxin Gene *(stn)* which is commonly found among many *Salmonella* serovars. It has been detected in all strains *of Salmonella*. Due to its specificity and conservation in *Salmonella* Enterica Serotypes, the *stn* gene was chosen [22,23]. In *Salmonella* strains, the *stn* gene exhibits high nucleotide sequence homology but the little similarity to its corresponding gene in other closely related enteric bacteria. In addition, *stn* gene amplification has been reported to be effective in detecting 52 strains of *S. Enterica* and two strains of *Salmonella Bongori* without cross-reactivity to other more common intestinal strains [24].

In Egypt, data on the potential contribution of aquatic environments to *Salmonella* transmission are limited. Therefore, this study was designed to discover the prevalence of *Salmonella* among wild migratory and domestic birds in Lake Qarun. The second step included tracing the epidemiological relationship between *Salmonella* isolates from aquatic environments and those from other avian hosts using sequencing and phylogenetic tree analysis.

## Materials and Methods

### Ethical approval

The Institutional Animal Care and Use Committee of the Faculty of Veterinary Medicine at Cairo University authorized all animal procedures (VET. CU. IACUC; approval no. Vet CU 28042021304).

### Study period and location

The study was conducted from October 2018 to January 2019. In El-Fayoum Province, Egypt, a surveillance study was conducted in the PA of Lake Qarun. We collected water and other aquatic samples during migration season from four migratory ­waterfowl migration route ponds where birds were easily seen and trapped. In addition, commercial poultry farms located in the same geographic areas were sampled.

**Figure-1:**
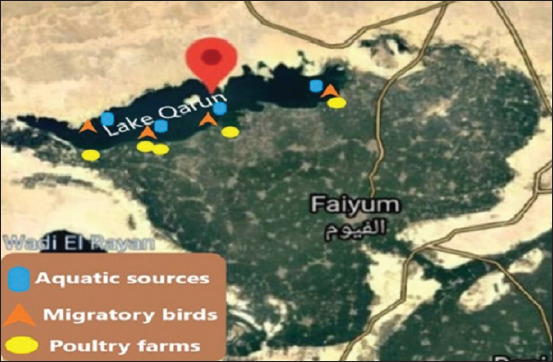
Sampling points and site selection of aquatic, migratory aquatic birds, and poultry samples. [Source: Google Earth].

Egypt’s Lake Qarun is the largest and most important lake in the country (Figure-1) [25]. Qarun PA is 1354 km^2^ in size. The water is now collected by two main drains, the El-Bats and El-Wadi drains, to provide agricultural drainage. Migration birds visit this wetland [7] because it is a significant wetland. In addition, human activity is present inside the PA. Agricultural land and, more recently, and fish farming are common on the southern shore of Lake Qarun, on the PA frontier.

### Sample collection

A total of 90 fecal samples from migratory wild birds and poultry chicken were collected, In addition to 222 samples from aquatic sources (water, fish, and crustaceans as the following:

#### Migratory wild birds

From October 2018 to January 2019, wild birds were trapped. Forty-five fecal samples from Lake Qarun in Fayoum Governorate yielded 20 northern shovelers (*Spatula clypeata*), eight green-winged teals (*Anas cardolinesis*), four mallard ducks (*Anas platyrhynchos*), and 13 Eurasian coots (*Fulica atra*). Waterfowl migrants have been caught using modified traps during winter migration. The trapped birds were kept in sterilized cages in a dark environment to minimize tension. A sterile swab was used to collect fecal samples. In the bacteriological processing laboratory, the swabs were placed in sterile saline (0.9% sodium chloride) and stored in an icebox after the birds were released.

#### Aquatic sources (water, finfish, and crustacean)

We collected 12 water samples from four sites (Figure-1) from the banks of Bats, Shakshuk village, Valley Bank, and Egypt for reconstruction by the bank of Bats. A sterilized 250-mL glass bottle was inverted below the water’s surface to obtain three samples from each site. Within an icebox surrounded by ice gel packs, samples were labeled and transported directly to the laboratory. In addition, 35 Nile tilapia finfish (*Oreochromis niloticus*) were collected with samples of water from Lake Qarun (Figure-1). We received fresh fish samples packaged in double sterile polyethylene bags and shipped them in a dry, clean icebox to be analyzed for clinical, postmortem, *Salmonella*, and bacterial isolation. Clinical evaluations were performed on all fish samples obtained according to Amlacher [26]. A sterile bacteriological swab was used to collect muscle and intestinal contents, in addition to surface swabs, from each fish sample before it was pre-enrichment in buffered peptone water (BPW). In addition, 175 samples of brine shrimp (*Artemia salina*) were collected from various locations along Lake Qarun.

#### Poultry farms

Five poultry farms located near the same geographic area of the survey zone provided 45 samples of chicken droppings. Each farm provided nine samples. Pre-enriched samples were kept on ice and transported in BPW from Oxoid Limited (Hampshire, England).

### Isolation and identification of Salmonella in different samples

According to the International Organization for Standardization [27], *Salmonella* was isolated and identified. In brief, cloacal swabs from wild birds and a farm were incubated at 37°C for 16-18 h in BPW (Oxoid CM509, Oxoid Limited, Hampshire, England). A membrane filtration system (BioMerieux^®^, Marcy-l’Étoile, France) was used to filter water samples into sterile filter membranes (0.45 m) that retained bacteria. To pre-enrich the filtrate, the filter membranes were transferred into BPW using a vortex [BRP^®^-H05W-F; American Public Health Association (APHA), Washington, USA] [28]. Swabs and muscles and intestinal contents from each fish were taken aseptically, inoculated into a sterile container, homogenized with 9-mL BPW, and incubated at 37°C for 16-18 h. As described by the APHA [29], shrimp samples were pooled by pooling every 3-5 shrimps. Then, the shrimp samples were homogenized with 10-mL sterile BPW in a stomacher. During the 16-18-h incubation period, the samples were pre-enriched with buffered peptone broth. After that, 0.1 mL of the pre-enriched culture was transferred to Rappaport-Vassiliadis (Oxoid Limited, Hampshire, England) and incubated at 42°C. For each enriched broth, 10-μL cultures were streaked on xylose lysine deoxycholate (XLD) agar (Difco; BD, New Jersey, USA) and incubated at 37°C for 24 h. We assessed the presence of typical *Salmonella* colonies on XLD agar plates. A pure isolate culture of bacteria was obtained by spreading aseptically various selective agar onto freshly prepared nutrient agar plates. Conventionally, colonies were subjected to biochemical tests and verified using the RapID ONE system (Thermo Fisher Scientific, Massachusetts, USA).

### Molecular identification of *Salmonella*

Conventional polymerase chain reaction (PCR) was used to detect virulence genes (i.e., *invA* and *stn*) using the primers listed in Table-1 [22,30]. The PCR amplification of the *invA* gene was performed using EmeraldAmp^®^ GT PCR Master Mix (Takara Bio Inc., Shiga, Japan), according to Rahn *et al*. [30]. Then, the PCR amplification of the *stn* gene was performed using EmeraldAmp^®^ GT PCR Master Mix (Takara Bio Inc, Shiga, Japan) at the following

**Table-1:** Primers of virulence genes (*invA* and *stn*).

Target gene	Primer	Primer sequence	PCR product (bp)	Reference
*InvA*	*InvA*-F	GTGAAATTATCGCCACGTT CGGGCAA	284	[30]
	*InvA*-R	TCATCGCACCGTCAAAGGAAGGAACC-		
*Stn*	*Stn*-F	TTG TGT CGC TAT CAC TGG CAA CC	617	[22]
	*Stn*- R	ATT CGT AAC CCG CTC TCG TCC		

Conditions: Initial denaturation at 95°C for 3 min and then 25 amplification cycles of denaturation, annealing, and extension at 94°C, 59°C, and 72°C, respectively, for 1 min in each step, followed by a final extension at 72°C for 10 min. PCR products amplified by PCR were electrophoresed on 1.5% agarose gels [22].

### Sequencing and phylogenetic analysis

Five isolates were purified using a QIAqick Gel Extraction Kit (Qiagen, Hombrechtikon, Switzerland); sequencing was performed at Promega Lab Technology (Madison, USA) using forward and reverse primers (Table-1). In the GenBank database, a sequence for the *stn* gene has been deposited under accession numbers listed in Table-2. We evaluated the sequences of the sequenced genes against those available from public databases on the NCBI BLAST server. In BioEdit (version 7.0.1.4; Informer Technologies, Inc., California, USA), *stn* gene sequences were aligned using ClustalW. MEGA (version 7) has been used to perform phylogenetic analysis using neighbor-joining. Then, 1000 bootstrap replicates were used to estimate the consensus tree.

**Table-2:** The different *Salmonella* sequences obtained from Lake Qarun.

No. of isolates	Isolation source	Accession number
Isolate 1	Water sample	MZ927032
Isolate 2	Fish	MZ927033
Isolate 3	Chicken	MZ927034
Isolate 4	Chicken	MZ927035
Isolate 5	Wild bird	MZ868594

### Statistical analysis

The data were analyzed using PASW Statistics (version 18.0; SPSS Inc., Chicago, IL, USA). The relationship between the occurrences of *Salmonella* in different aquatic samples was determined using the chi-square test (χ^2^). p*<*0.05 was used to denote statistical significance.

## Results

From various environmental sources, the prevalence of *Salmonella* was compared. The prevalence of *Salmonella* is shown in Table-3; the highest percentage was recorded from lake water 58.3% (7/12), followed by isolation from migratory aquatic birds 13.3% (6/45). In contrast, *Salmonella* was found in 6.6% (3/45) of the commercial poultry sources examined and 4.3% (3/70) from different aquatic organisms mainly isolated from Nile tilapia fish. Statistically, a significant difference was observed in the distribution of *Salmonella* among various samples (p<0.0001).

**Table-3:** Prevalence of *Salmonella* in different sources collected from Lake Qarun.

Environmental samples	No. of examined samples/pools	Prevalence of *Salmonella*		Chi-square value and significance

		No. of positive samples	%	
Lake water	12	7	58.3^a^	χ^2^=31.68, p<0.0001*
Migratory aquatic bird	45	6	13.3^b^	
Commercial Broiler	45	3	6.6^c^	
Fish and brine shrimp	70	3	4.3^c^	
Total	172	19	11.04	

^a,b,c^ *Different superscripts indicate significance at p<0.05

With regard to the prevalence of *Salmonella* isolated from avian sources (migratory wild birds and commercial broiler chicken, the data in Table-4 indicate no significant difference in prevalence between *Salmonella* occurrence and the types of birds (migratory birds and broilers) collected near the aquatic ecosystems examined at Lake Qarun. The p-value was 0.29, and the Chi-square value was 1.11. In migratory aquatic bird species that were sampled, *Salmonella* was isolated from 23.1% (3/13) of Eurasian coot (*F. atra*), 12.5%, (1/8) of green-winged teal (*A. cardolinesis*), 10% (2/20) of northern shoveler (*S. clypeata*), and 0% (0/4) of mallard duck (*A. platyrhynchos*). In total isolation from migratory aquatic birds, 13.3% (6/45), which is higher than *Salmonella* isolation from the commercial boiler chicken 6.6% (3/45).

**Table-4:** Prevalence of *Salmonella* in the examined cloacal samples collected from wild and domesticated bird species.

	Bird species		No. of examined cloacal samples	Prevalence of *Salmonella*		Chi-square value and significance
	
	Common name	Scientific name		No.	%	
Migratory wild birds	Northern shoveler	*Spatula clypeata*	20	2	10	χ^2^=1.11, p=0.29
	Green-winged teal	*Anas cardolinesis*	8	1	12.5	
	Mallard duck	*Anas platyrhynchos*	4	0	0	
	Eurasian coot	*Fulica atra*	13	3	23.1	
Total Migratory wild birds			45	6	13.3	
Commercial broiler	5 broiler farms	*Gallus gallus domesticus*	45	3	6.6	

The p-value is 0.29. The result is not significant at p<0.05

As shown in Table-5, *Salmonella* isolates in Lake Qarun had significant differences in the prevalence between sources (i.e., cultured tilapia, brine shrimp, and water). In the study where χ^2^=13.2 and p=0.00027, *Salmonella* isolates 8.6% (3/35) were mainly found on the external surface and intestines of fish, as they were never isolated from the muscles of fish nor from shrimps. The prevalence of *Salmonella* in water samples was high, 58.3% (7/12).

**Table-5:** Prevalence of *Salmonella* in fin fish, brine shrimp, and water samples collected from Lake Qarun.

	Aquatic source	Specimens	No. of positive samples/pools	*Salmonella* isolates (%)	Chi-square value and significance
	35 Nile tilapia (*Oreochromis niloticus*)	35 External surfaces	2	3/35 (8.6%)^b^	χ^2^=13.2, p=0.00027*
		35 Muscle	0		
		35 Intestine	1		
	175 brine shrimp (*Artemia salina)*	35 pools (5 artemia for each pool)	0	0%	
	12 water	12	7	7/12 (58.3%)^a^	
Total	222 aquatic samples	152 specimens and pools	10	10/222 (4.5%)	

* ^a,b^the result is significant at p<0.05

**Figure-2:**
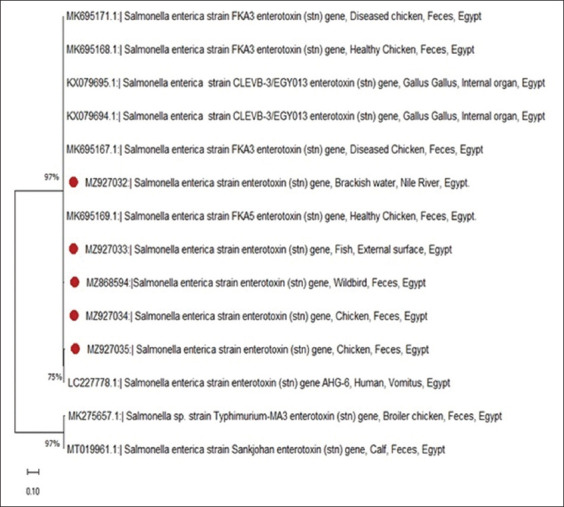
Neighbor joining phylogenetic tree with nucleotide sequences of 5 stn genes of *Salmonella* spp. The isolates used in the current study labeled with red color. The analysis was generated using neighbor-joining method with MEGA X software version 10.2.0.

Compared with migratory wild birds and lake water, sequence analysis and comparison of the *stn* gene showed a high degree of homology between them (Figure-2). These sequences clustered with isolated sequences retrieved from the GenBank databases that originated from humans, wastewater, and surface water.

## Discussion

In Egypt, foodborne pathogens, such as *Salmonella*, pose substantial health risks [31]. According to the World Health Organization [32], *Salmonella* species account for the third-highest number of deaths among the 31 etiological agents of diarrhea and/or infectious diseases. Approximately 95% of human salmonellosis cases can be traced back to the consumption of foods containing *Salmonella*, such as meat, poultry, eggs, milk, and seafood [33].

Fifteen percent of Egyptian commercial fishing is conducted in Egyptian lakes. Industrial and agricultural effluents contaminate the lakes. The spread of many diseases in the lake is caused by microbial pollution, which is one of the most harmful types of water pollution. In this regard, assessing microbial contamination in Egyptian lakes is imperative, particularly Lake Qarun, which is considered an important source of freshwater and salts for the province of Fayoum [34].

Fish and other aquatic animals were investigated in this study as zoonotic carriers of *Salmonella*, as they pose a risk to humans. Poor sanitation and improper disposal of human and animal waste can all contribute to the introduction of *Salmonella* into aquatic environments [35]. Thus, environmental samples from around Lake Qarun, migratory aquatic birds, poultry carcasses, and finfish were tested for *Salmonella*, with 58.3%, 13.3%, 6.6%, and 4.3% of *Salmonella* isolated from the samples, respectively (Table-3). We found that aquatic environments are the major reservoirs of *Salmonella*, which was also supported by Bianco *et al*. [36]. Thus, fishery products are major carriers of foodborne pathogens. A high percentage of *Salmonella* isolated from Lake Qarun (58.3%) was described in a study conducted by Upadhyay *et al*. [37], who noted that Lake Qarun was the largest reservoir of agricultural and sewage drainage in Fayoum and fish farms surrounding the lake. In addition, Al-Afify *et al*. [34] have reported that the discharge of agricultural and municipal sewage into Lake Qarun significantly affects the quality of its water. Moreover, the lake water quality index values were low, making irrigation and aquatic life guidelines unfeasible. *Salmonella* is often isolated from water [38], which serves as a bacterial reservoir and may facilitate the transmission of the bacteria [39]. *Salmonella* can be continuously released into the environment by infected humans, farm animals, pets, and wildlife. Moreover, *Salmonella* can survive in septic tanks for up to 15 days despite efforts to contain and sanitize waste [40]. Water contamination, particularly with animal pathogens that are also pathogenic to humans, might put those handling in close contact with the water and fish at the facility at risk of illness [41].

Among other sources of marine and freshwater contamination were aquatic migratory birds, which are found in the same area. Wild and migratory birds usually cross national boundaries using many habitats, including marshes, grain stores, pastures, and other water bodies [42]. The presence of these diseases will pose public health hazards along bird migration routes, which will act as new foci of emerging and reemerging bacterial diseases [43]. As shown in Table-4, the occurrence of *Salmonella* in 45 fecal samples of migratory birds collected from October 2018 to January 2019 from the PA of Lake Qarun was 13.3%. This finding was higher than the result obtained by National Institute of Allergy and Infectious Diseases [44] and Craven *et al*. [45], who reported percentages of 10.75% and 10.6%, respectively. In addition, studies by Awadallah *et al*. [46] and Kobayashi *et al*. [47] have shown lower infection rates for *Salmonella* (5.8% and 7.4%, respectively). In this study, *Salmonella* was found in higher percentages owing to the consumption of polluted water due to leaks of human sewage into water canals. The availability of human waste disposal sites was previously suggested by Vlahović *et al*. [48], who observed that enteric bacteria are more likely to circulate through migratory birds when human waste is available. In this way, wild bird feces are potentially contaminated with some pathogenic bacteria, posing zoonotic risks. Moreover, waterfowl that migrate and live in water areas thrive on zooplanktonic copepods and chironomids, which can remain in the gut for a long time. Moreover, these viable zooplanktons can attach to external surfaces of waterfowl.

Both findings indicate that waterfowl disperse bacterial pathogens from an aquatic habitat to other environments [49-52]. Fish pond manure is used not only to increase output by reducing carbon-to-nutrient ratios but also in other ways [53]. However, animal manure, classified as a hazardous organic matter, poses a risk to the aquatic environment [20]. It is possible that integrated livestock–fish farming introduces pathogenic bacteria to pond water and sediments through manure [54-55]. Various pathogenic microorganisms were detected in manure, besides the typical microflora of animal intestines, as determined by microbiological analysis [56]. Infections are transmitted more readily through manure and water environments where pathogens persist. Depending on the type of manure, heat, pH, oxygen content, level of ammonia, and presence of competing organisms, zoonotic pathogens can survive in such an environment for up to 4 months [57]. Table-4 shows that *Salmonella* was isolated from 45 fecal samples (6.6%) of chicken from five farms. Various sources of infection might have affected domesticated broilers, which may have spread to contaminate aquatic facilities. Hence, using chicken manure in fish farming could pose a potential public health risk to humans [18].

A major source of non-typhoid *Salmonella* is the feces of domesticated and wild animals, which serve as a vehicle for the transmission from animals to humans. These microorganisms can transfer from an infected animal or human to food preparation areas, where they consequently proliferate in food items due to improper storage temperatures, inadequate cooking, and/or cross-contamination [58]. Furthermore, considering that many foods can be consumed instantly, controlling and reducing *Salmonella* levels is crucial. To avoid cross-contamination in consumer kitchens, bacterial control is essential even if the food is cooked before consumption.

In Table-5, *Salmonella* isolated from the intestines and external surfaces of fish indicate fecal contamination of the water where the fish were caught, which can pose a threat to the public. In addition to bacteria, contaminants introduced by animal and human wastes during processing or production chain preparation can contaminate seafood [59]. *Salmonella* is more likely to be contracted when poorly maintained hygiene standards are present and individuals consume raw seafood from water tainted with sewage or feces [60]. Fish and fish products contaminated by *Salmonella* are commonly responsible for food poisoning outbreaks in Egypt, with clinical signs, including enteritis and systemic illness [61]. Thirty serovars have been identified in aquatic environments, illustrating the likelihood and diversity of contamination of fish and shellfish [62]. Unlike high isolation percentages from water and fish, no isolates were found from crustacean fish.

To determine the epidemiological relationship between aquatic environment, wild birds, and chicken, phylogenetic analysis using *stn* genes isolated from these sources was performed. The results of our study and previous studies indicate that *stn* is an appropriate target gene for the detection of *Salmonella* in biological samples.

Surprisingly, all the study sequences were found in the same cluster (Figure-2), which indicates strong relation between these isolates. In addition, *stn* genes sequences showed higher nucleotide identity percent between the sequences recovered from our study isolates and human, indicating a significant public health concern. This finding indicated the dissemination of this gene between different sources. Moreover, this confirmed the role of wild birds in the environmental dissemination of *Salmonella* as they considered a potential spreader of zoonotic bacterial pathogen through the ability to migrate long distances in short periods of time and its effect on the environment. Therefore, *Salmonella* infections in the aquatic environment may be associated with *Salmonella* infections originating from migratory waterfowl. *Salmonella* may survive in an environment with a broad range of pH (4.05–9.5) and can multiply in a broad range of temperatures (7–48°C) [63]. It can also be noticed the close relationship between the chicken sequence and the fish sequence (Figure-2). This relation highlights the scenarios of using chicken manure (CM) in fish farms and its public health implication. Integrated livestock-fish farming may be associated with the risk of contamination of pond water and sediments with pathogenic bacteria from the manure [34].

## Conclusion

Migratory aquatic birds could serve as a vector for cyclic *Salmonella* transmission among aquatic animals, poultry, humans, and wildlife. Based on sequence comparisons, migratory waterfowl and water isolates cluster with human and aquatic isolates from the GenBank database. Moreover, a close relationship exists between chicken and fish sequences. Aquatic environments, particularly lake water, are natural reservoirs for *Salmonella*. Consequently, in addition to the ongoing monitoring of lake water quality and pollution mitigation with hygienic guidelines and biosecurity protocols in animal production and industry, these measures might be useful in combating this problem

## Authors’ Contributions

HSK and ZSA: Isolated *Salmonella* strains from fish and birds. HSK and EAE: Compared strains, interpreted data, and drafted and revised the manuscript. FA and EMI: Conceived and designed the study and revised the manuscript. All authors read and approved the final manuscript.
